# Breakthrough in High-Efficiency Photocatalytic Degradation of Acebutolol by Advanced Binary CeO_2_–MnO_2_ Oxide System

**DOI:** 10.3390/molecules29122854

**Published:** 2024-06-15

**Authors:** Muthuraj Arunpandian, Tae Hwan Oh, Ganesan Sriram

**Affiliations:** School of Chemical Engineering, Yeungnam University, 280 Daehak-Ro, Gyeongsan 38541, Republic of Korea; muthurajarunpandian1523@yu.ac.kr

**Keywords:** binary, morphology, efficient, acebutolol, TOC, stability

## Abstract

The sustainable catalytic efficacy of transition metal oxides (TMO) and rare earth element-based oxides positions them as pivotal materials for effectively treating contaminated wastewater. This study successfully synthesized a series of Ce@MnO_2_ photocatalysts using a straightforward hydrothermal method. These photocatalysts were thoroughly characterized for their optical properties, structural morphology, and phase purity. Among the synthesized materials, the Ce@MnO_2_ (40:60) exhibited the highest photocatalytic activity for the degradation of Acebutolol (ACB), achieving a remarkable degradation efficiency of 92.71% within 90 min under visible light irradiation. This superior performance is attributed to the increased presence of active species and the efficient separation of photogenerated carriers. Additionally, the photocatalytic reaction mechanism was elucidated, highlighting the catalyst’s surface charge properties which significantly enhanced performance in a solution with pH 8. The outstanding photo-response in the visible spectrum renders this method not only cost-effective but also environmentally benign, presenting a promising approach for large-scale water purification.

## 1. Introduction

In recent years, pharmaceutically active chemicals have emerged as critical environmental pollutants due to their persistent introduction and enduring presence in aquatic ecosystems [1]. These compounds have been detected in sewage effluents, surface and groundwater, and even potable water. Once administered, these substances, along with their metabolites and unchanged original forms, are frequently discharged into sewage systems [2,3]. Consequently, pharmaceutical residues are now commonly found in various water samples, including wastewater treatment plant effluents, surface water, and drinking water [4,5,6]. Of particular concern is Acebutolol (ACB), a beta-blocker, which can lead to significant adverse effects even at low concentrations. ACB is not fully metabolized within the human body and is excreted unchanged, continuously entering sewage streams through the disposal of unused or expired medications and pharmaceutical discharges, thereby impacting both aquatic and terrestrial ecosystems [7,8].

Given the potential detrimental effects of ACB at trace levels in aquatic environments, there is an ongoing effort to develop new and selective analytical methods for its complete removal. Several techniques have been explored for the degradation of ACB, yet these methods often face significant limitations such as high costs, time-consuming processes, and the necessity of complex sample preparations involving various derivations, extractions, and purification steps. Among these methods, photocatalysis stands out as the most promising approach for wastewater treatment due to its simplicity, cost-effectiveness, and environmental friendliness [9,10]. Consequently, research has increasingly focused on identifying robust and sustainable photocatalytic systems for ACB degradation.

Recent advancements in metal-based bifunctional photocatalysts have aimed to enhance efficiency while reducing costs. Efforts have centered on developing highly efficient catalysts to facilitate degradation processes. Various materials have been investigated as cathode catalysts, including noble metals (Au, Pt, Pd, Ru, Ir), transition metal oxides [10,11,12,13,14,15,16,17], carbon-based materials [18,19,20,21], perovskite-type oxides [22,23,24], and heterogeneous composites [25,26]. Transition metal oxides, in particular, offer advantages such as low cost, straightforward synthesis, and high catalytic activity [27,28]. Among them, CeO_2_ and MnO_2_ have been extensively used in catalytic applications due to their excellent performance. MnO_2_ is especially promising due to its high theoretical capacitance (1370 F/g), natural abundance, environmental friendliness, and low cost [27]. Recent studies have demonstrated the effectiveness of MnO_2_-based materials in decomposing industrial dye effluents, with their catalytic performance in photocatalysis being significantly influenced by the rate of reabsorption of photogenerated electron–hole pairs. The surface morphology and crystal structure are important factors determining their degradation performance [28,29,30,31,32]. Similarly, cerium (Ce)-related catalysts enhance efficiency by promoting oxygen storage and mobility, forming surface and bulk vacancies, and improving redox properties when combined with other metals [33,34,35].

This study focuses on the synthesis and application of Ce@MnO_2_ nanocomposites for the photocatalytic degradation of ACB. Using a hydrothermal method, we created Ce@MnO_2_ nanostructures and extensively analyzed their physicochemical properties. This research marks the first instance of employing Ce@MnO_2_ for ACB degradation, and the results demonstrate a significantly enhanced photocatalytic efficiency, providing a viable solution for mitigating pharmaceutical pollutants in water.

## 2. Results and Discussions

### 2.1. Crystal Structure Analysis

**Figure 1:**
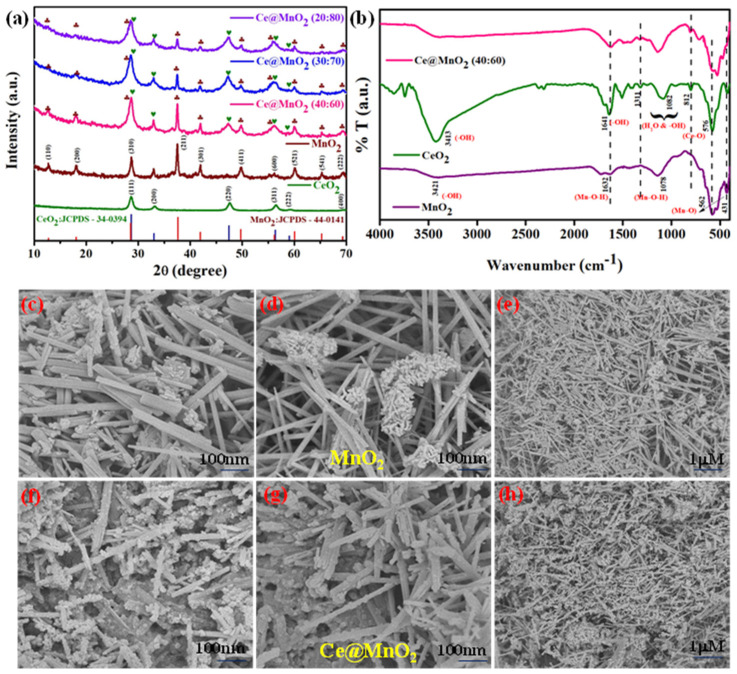
Crystalline structure and morphology analysis: (**a**) X-ray powder diffraction patterns of the CeO_2_, MnO_2_, Ce@MnO_2_ (40:60), Ce@MnO_2_ (30:70) and Ce@MnO_2_ (20:80) photocatalysts, (**b**) FTIR analysis of MnO_2_, CeO_2_ and Ce@MnO_2_ (40:60), and FE-SEM analysis: (**c**–**e**) MnO_2_, (**f**–**h**) Ce@MnO_2_ (40:60) nanocomposite.

Nanomaterials were examined for their crystalline size and phase structure using the X-ray diffraction (XRD) method. Figure 1a shows the XRD spectra of MnO_2_, CeO_2_, and binary CeO_2_-MnO_2_ at various dose levels, in addition to the host materials. The diffraction peaks may be seen in the figure at certain angles of viewing: 12.71°, 18.01°, 28.64°, 32.98°, 37.58°, 42.05°, 49.82°, 56.23°, 60.02°, 65.31°, and 69.45°. The diffraction from the (110), (200), (310), (400), (211), (301), (411), (600), (521), (541), and (222) crystallographic planes corresponds to these peaks. All of the diffraction peaks, according to the analysis, are exclusively associated with a tetragonal phase of α-MnO_2_ (standard JCPDS card no: 44-0141) [32], which has lattice constants of a = 0.9784 and c = 0.2863 nm. Due to the absence of any other phases or planes in the diffraction patterns, the synthesized pure α-MnO_2_ exhibits a high degree of phase purity, comparable to the equivalent α structure. Diffraction peaks at 28.71°, 33.18°, 47.62°, 56.37°, 59.08°, and 69.52° are suggestive of pure cerium oxide (CeO_2_) with a cubic fluorite structure, according to the JCPDS data (JCPDS No. 34-0394) [36]. In that order, these peaks correspond to crystal planes (111), (200), (220), (311), (222), and (400), correspondingly. The successful fabrication of CeO_2_-MnO_2_ nanocomposite was prepared by the mixed metal oxides of CeO_2_ and a α-MnO_2_ matrix.

### 2.2. Fourier Transform Infrared Spectroscopy

The vibrational atom modes of the MnO_2_, CeO_2_, and CeO_2_-MnO_2_ (40:60) nanocomposites were confirmed by Fourier Transform Infrared (FTIR) analysis, as shown in Figure 1b. The prominent peak detected at 3421 cm^−1^ in the MnO_2_ spectra is ascribed to the stretching vibrations of O–H bonds, indicating that MnO_2_ is in a hydrated condition. The absorption bands observed at 1632 and 1078 cm^−1^ are ascribed to the bending vibrations of the O–H bonds in combination with the Mn atoms. The peaks detected at 562 and 431 cm^−1^ are ascribed to the vibrations of Mn–O bonds [37]. The FT-IR spectrum of CeO_2_ displays a peak at 576 cm^−1^, which is attributed to the Ce-O vibration. The spectrum of the CeO_2_ nanoparticles, as shown in Figure 1b, displays absorption peaks at distinct wavenumbers: 3329, 1641, 1311, 1082, and 812 cm^−1^. The bands observed in the spectrum are attributed to the stretching modes of the water and hydroxyl groups, as well as the vibrations related to the incoordination of the adsorbed NO_3_^−1^ ion [38,39]. The existence of the Ce@MnO_2_ nanocomposite is apparent based on the distinct observation of two significant bands at 432 and 553 cm^−1^. The bands correspond to the division of the vibrational band linked to the Ce-O or O-Mn-O bond, respectively.

### 2.3. Morphology Structure Analysis

#### Scanning and Transmission Electron Microscopy Analysis

**Figure 2:**
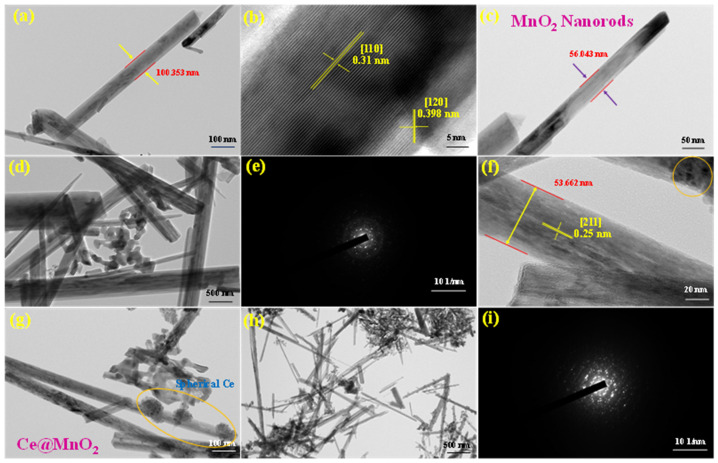
HR-TEM images of (**a**–**d**) MnO_2_ at various magnification ranges. (**e**) Selected area electron diffraction (SAED) pattern of the resultant MnO_2_; (**f**–**h**) display the HR-TEM images of Ce@MnO_2_ nanocomposite and (**i**) shows the selected area electron diffraction (SAED) pattern of the resultant Ce@MnO_2_ (40:60) nanocomposite.

The surface morphology of the as-prepared Ce@MnO_2_ NCs was assessed using FE-SEM analysis. The photos in Figure 1c–h show the low (1 µm) and high magnification (100 nm) field emission scanning electron microscopy (FE-SEM) images of MnO_2_ and Ce@MnO_2_ nanocrystals (NCs). These images reveal a structure consisting of randomly twisted and interlinked nanorods that resemble spherical shapes, with clean and smooth edges. Additionally, HR-TEM experiments were conducted to obtain precise morphological data on the Ce@MnO_2_ NCs. The high-resolution transmission electron microscopy (HR-TEM) images shown in Figure 2a–d provide definitive evidence of the nanorod-like shape and loosely packed nanostructure of MnO_2_. Based on the information provided in Figure 2f–h, it can be observed that the nano spherical-like cerium oxide was effectively integrated onto the surface of MnO_2_. The nanorods have an average size of approximately 50-100 nm, with a corresponding thickness. The photogenerated carriers are better separated when CeO_2_ and MnO_2_ are close, improving the photocatalytic performance. The high-resolution transmission electron microscopy (HR-TEM) image in Figure 2f reveals clear lattice fringes with a lattice spacing of 0.25 nm, which can be attributed to the (211) plane of the tetragonal MnO_2_ phase. This observation is consistent with the X-ray diffraction (XRD) findings.

### 2.4. DRS-UV Analysis

**Figure 3:**
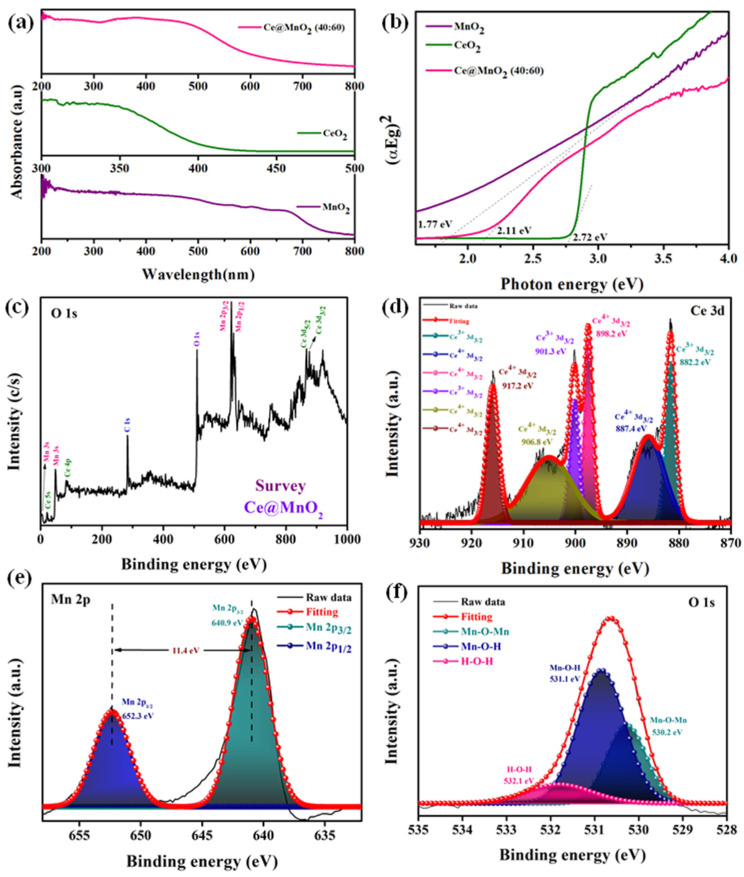
Optical properties analysis: (**a**) DRS-UV spectra of various catalysts and (**b**) corresponding Tauc plots of various as prepared nanocatalysts, (**c**–**f**) XPS spectrum of the Ce@MnO_2_ (40:60) nanocomposite, (**c**) survey spectrum, (**d**) high-resolution spectra of Ce 3d, (**e**) Mn 2p, and (**f**) O 1s elements.

The absorption parameters of virgin MnO_2_, CeO_2_, and the Ce@MnO_2_ composite were measured using UV-Vis diffuse reflectance spectroscopy, with a specified concentration of CeO_2_ and MnO_2_ (Figure 3a). The absorption edge of pure MnO_2_ occurred at a wavelength of 780 nm in the visible area. The pristine CeO_2_ exhibited an absorption edge at a wavelength of 424 nm, which is associated with intrinsic band gap absorption. The Ce@MnO_2_ composite exhibited a greater absorption edge compared to both pure CeO_2_ and MnO_2_. The red-shift indicates increased absorption of visible light and enhanced generation of electron–hole pairs by the composite material. The sample’s band gap energy (Eg) can be determined by analyzing the graph depicting the correlation between the square root of (αhν) and the photon energy (hν). The Tauc plot equation is utilized to ascertain the band energy value of materials in their initial state of existence:

αhν = A (hν − Eg)^2^ (1)

In this context, the symbol α indicates the absorption coefficient, hν refers to the light source, A is a numerical value that represents the proportionality constant, and Eg is the energy value. The linear sections of the plots of (hν) against (αhν)^2^ were extrapolated to determine the energy bandgap of the samples. The values of MnO_2_, CeO_2_, and Ce@MnO_2_ were determined to be 1.77, 2.72 and 2.11 eV, respectively, as shown in Figure 3b [40,41]. Furthermore, based on the principle of light reflection spectrum, the composite Ce@MnO_2_ often exhibits a lower value compared to the host MnO_2_. The surface of the composite enhances the light absorption efficiency of Ce@MnO_2_. This has been confirmed through calculations of light absorption using the UV-Vis diffuse reflectance spectrum in Figure 3b. The Ce@MnO_2_ exhibited heightened light absorption within the visible light spectrum. It has been verified that Ce@MnO_2_ has a greater capacity for light absorption compared to the host materials MnO_2_ and CeO_2_. These data are highly significant for enhancing photocatalytic efficiency.

### 2.5. XPS Analysis

The valence states of the elements in the Ce@MnO_2_ nanocomposite were evaluated and a thorough examination of the components was carried out using X-ray photoelectron spectroscopy (XPS) (Figure 3c). The presence of Ce, Mn, and O components was confirmed by the survey spectrum, as shown in Figure 3c, which validates the synthesis of the nanocomposite. Figure 3d shows the high-resolution spectra of Ce 2p as a result of the X-ray photoelectron spectroscopy (XPS) investigation. The 882.2 and 901.3 eV Ce^3+^ and 887.4, 898.2, 906.8, and 917.2 eV Ce^4+^ peaks were identified in this study. Other XPS spectra published in the literature [42] agree with the peak locations. The two distinct peaks at 640.9 and 652.3 eV, which correspond to Mn 2p_3/2_ and Mn 2p_1/2_, respectively, may be seen in Figure 3e [43]. As is typical in MnO_2_, the energy gap between the 2p_3/2_ and 2p_1/2_ orbitals of Mn is around 11.4 eV [44]. Figure 3f shows that the O 1s peak was deconvolution, yielding three distinct peaks at approximately 530.2 eV, 531.1 eV, and 532.1 eV. These peaks can explain the presence of structurally linked hydroxide (^−^OH) groups and O_2_^−^ anions, namely Mn-O-Mn and Mn-O-H [45]. The X-ray photoelectron spectroscopy (XPS) study verifies the effective synthesis of Ce@MnO_2_ nanocomposites.

### 2.6. BET Analysis

**Figure 4:**
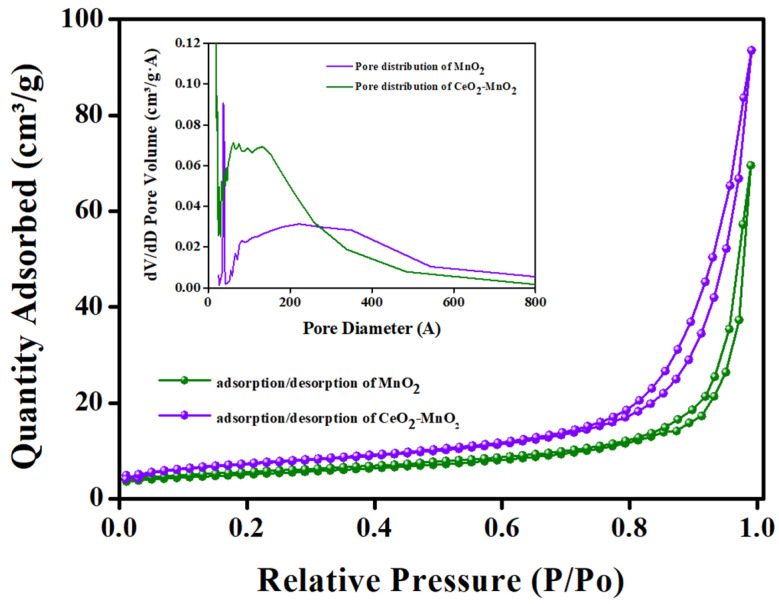
Surface area analysis: The N_2_ physical adsorption isotherms and (inset) pore size distribution of MnO_2_ and CeO_2_-MnO_2_ nanocomposites.

The active specific surface area of the MnO_2_ and CeO_2_-MnO_2_ samples was obtained using BET analysis. This experiment utilized the N_2_ adsorption–desorption isotherm curve depicted in Figure 4. The MnO_2_ and CeO_2_-MnO_2_ nanostructures were found to have surface areas of 17.59 and 25.56 m^2^g^−1^, respectively. Furthermore, an increased surface area results in a greater number of active sites, hence enhancing catalytic activity. The pore-size curves of these different nanostructures are displayed in the inset of Figure 4. The nanostructures MnO_2_ and CeO_2_-MnO_2_ possess pore diameters of approximately 38.1 nm and 22.6 nm, respectively.

### 2.7. Photocatalytic Degradation Performance of ACB

**Figure 5:**
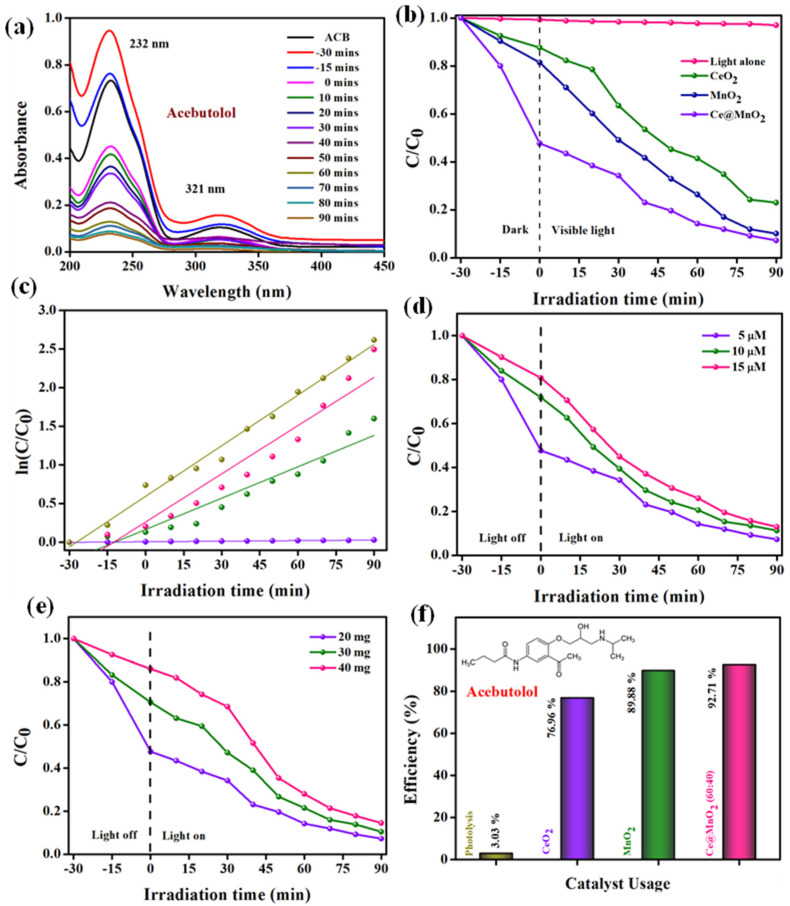
Photodegradation of ACB under various optimized parameters: (**a**) Absorbance spectra for the degradation of Acebutolol, (**b**) different catalyst usage for ACB degradation (catalysts: 20 mg/L, ACB conc.: 1 × 10^−5^ M, (**c**) kinetics parameter for ACB drug degradation under various catalyst usage (**d**) different concentration variation of ACB using, (**e**) different catalyst usage for ACB degradation, (**f**) degradation efficiency towards the degradation of ACB drug using various catalysts.

The degradation of the ACB drug through photocatalysis was seen by analyzing the changes in the primary absorption peak of ACB over time under UV-Vis spectra, in the presence of Ce@MnO_2_ NCs. Figure 5a illustrates the variation in absorption intensity of an aqueous suspension of ACB (10 μM) at 232 nm for a period of 0 to 90 min under visible light exposure, in the presence of Ce@MnO_2_ (40:60) at a concentration of 20 mg. The absorption peak at 237 nm and the smaller peaks at 321 nm, as shown in Figure 5a, gradually dropped and reached almost nothing after 70 min. This indicates that more than 92% of the ACB was destroyed by Ce@MnO_2_ after 90 min of exposure to visible light. Before the photodegradation reaction, control tests were conducted to observe the degradation of ACB in the absence of a catalyst (using only light). The results of these studies are shown in Figure 5b. In addition, the effectiveness of Ce@MnO_2_ NCs as a photocatalyst for breaking down ACB was compared to other catalysts including CeO_2_ and MnO_2_, and the results can be shown in Figure 5b. The results clearly demonstrate that Ce@MnO_2_ (40:60) exhibits a superior photo-degradation performance (92.71%) compared to CeO_2_ (76.96%) and MnO_2_ (89.88%) when tested against the ACB. The increased photocatalytic performance of Ce@MnO_2_ can be ascribed to its well-organized spherical nanorod structure, high crystallinity, and extensive active surface area.

The kinetics of the photodegradation of ACB with Ce@MnO_2_ were investigated using a pseudo-first-order kinetic equation, as shown in Equation (2).

ln(C_0_/C) = kt (2)

In this equation, C_0_ represents the initial concentration of ACB at time t = 0, C represents the concentration of ACB at different light irradiation times, k is the rate constant, and t represents the time. Figure 5c illustrates the relationship between the rate at which ACB degrades during light exposure and the duration of the irradiation. The calculated rates for CeO_2_, MnO_2_, and Ce@MnO_2_ were determined to be 0.0135, 0,0208, and 0.0219 min^−1^, respectively.

In addition, the influence of varied beginning ACB concentrations on its photodegradation was examined by applying Ce@MnO_2_ (40:60) NCs under the same conditions (catalyst dose of 20 mg and light source with the same conditions). The photodegradation efficiency of ACB was observed to decline with the quantities of ACB, as illustrated in Figure 5d. As a result of this, the absorption of charge carriers that are created by light may be inhibited when the concentration of ACB is enhanced. This occurs because the abundance of ACB molecules hinders the movement of charge carriers towards the surface of the catalytic material. Moreover, it is feasible that the ACB molecules, rather than the catalyst, might be the ones to take in a particular quantity of energy from the photons that are being created during the reaction. As a consequence of this, the catalyst produces a restricted quantity of hydroxyl radicals, which ultimately results in a reduction in the percentage of degradation that occurs as a consequence of the severe rivalry that exists among consumers. Throughout the degradation process, a diverse range of catalysts was employed under carefully optimized conditions. These conditions encompassed the optimal catalyst loading dosage and ACB concentration. The level of efficiency attained due to the degradation is illustrated in Figure 5f.

The photocatalyst dosage is a main factor in the photodegradation reaction. Figure 5e displays the different amounts of Ce@MnO_2_ NCs, ranging from 20 to 40 mg, while keeping the ACB concentration constant. The data demonstrate a continuous increase in the photodegradation effectiveness of ACB as the dose quantity is increased from 20 to 40 mg. However, the efficiency of degradation consistently falls after reaching 20 mg. The primary factor is that as the quantity of the catalyst is augmented, a greater number of reactive active sites are required for the photodegradation process, hence enhancing the degradation efficiency. However, excessive loading can lead to an accumulation of catalyst particles, impeding the access of reactive active sites to the catalyst surface. Furthermore, due to reduced light penetration and enhanced light dispersion, there was a significant decrease in visible light intensity.

**Figure 6:**
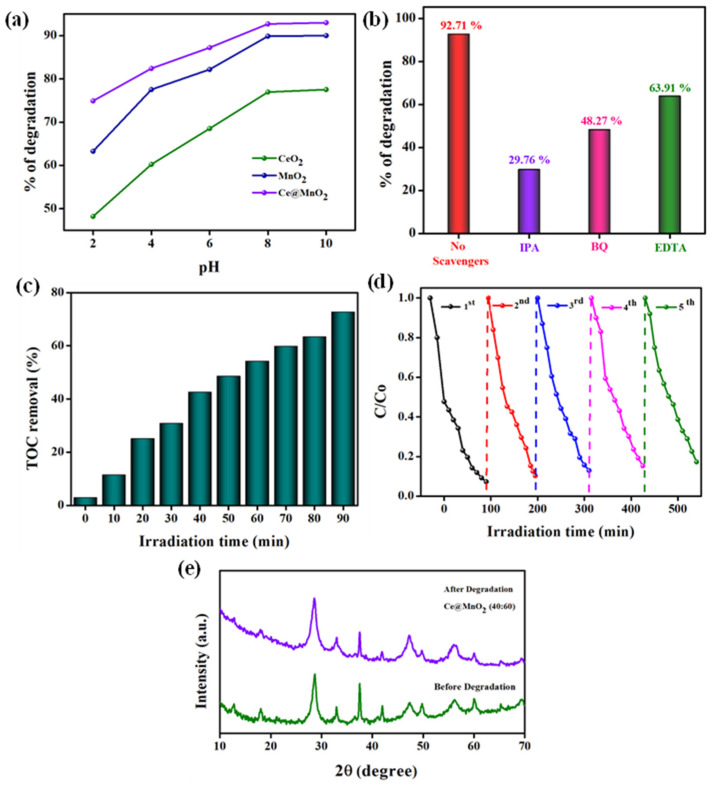
Photodegradation of ACB under various optimized parameters: (**a**) effect of pH on photocatalytic degradation of ACB, (**b**) influence of different scavengers towards the degradation of ACB drug, (**c**), TOC removal, (**d**) reusability of Ce@MnO_2_ NCs during five consecutive cycles for ACB degradation, (**e**) XRD patterns of Ce@MnO_2_ before (Green colour) and after 5 consecutive runs (purple colour).

The pH of the solution is an important factor in photocatalytic processes. The photocatalyst’s surface charge is significantly influenced by it [46]. Nevertheless, determining the impact of pH on the efficacy of the photocatalytic degradation process is a highly challenging endeavor due to the various reaction mechanisms involved. These mechanisms include hydroxyl radical attack, direct oxidation by positive holes, and direct reduction by electrons in the conducting band, all of which can contribute to the degradation of pollutants. The influence of solution pH on the rate of photocatalytic degradation in the presence of MnO_2_ and Ce@MnO_2_ materials was examined throughout a range of pH values from 2 to 10 (specifically, pH 2, 4, 6, 8, and 10), as depicted in Figure 6a. The pH of ACB solutions was modified using either NaOH or H_2_SO_4_ for acidic and basic conditions. The experimental results showed that the rate of photocatalytic degradation of ACB increased as the pH increased from 2 to 8. At pH levels over 8, the degradation efficiency of ACB remains constant. A high concentration of protons in an acidic solution (pH < 6) slows down the photodegradation of ACB, leading to a decrease in degradation efficiency. The degradation efficacy % reduces as the initial pH of the drug solutions decreases. The likely reason for this behavior is that once the initial pH of the drug solution lowers, Ce@MnO_2_ develops a positive charge on its surface. This positive charge can greatly enhance the adsorption of ACB, as ACB includes negatively charged sulfonate groups. This effect impedes the absorption of photons by the photocatalyst. As a result, the rate at which ACB degraded dropped. At an alkaline pH, the electrostatic contact between the surfaces of the catalyst and ACB results in a robust adsorption process, leading to an elevated degradation rate. The optimal pH for achieving the highest ACB degradation percentages was determined to be 8. At this pH, the degradation percentages were measured to be 85.2% and 90.7% for the MnO_2_ and Ce@MnO_2_ catalysts, respectively.

A well-known aspect of photocatalytic reactions is the production of active species, which include holes (h^+^), superoxide radicals (O_2_^•−^), and hydroxyl radicals (HO^.^). By conducting reactive species trapping tests, we were able to identify the main active species involved in ACB degradation. In simple terms, the sacrificial reagents for holes (h^+^), superoxide radicals (O_2_^•−^), and hydroxyl radical (HO^.^) were Ethylenediaminetetraacetic acid (EDTA), benzoquinone (BQ), and isopropanol (IPA), respectively [47]. Figure 6b shows that when isopropanol (IPA) was added, the photodegradation rate of acebutolol (ACB) dropped significantly to just 29.76%. Furthermore, photodegradation can still occur at rates of up to 48.27% and 63.91%, respectively, even when benzoquinone (BQ) and Ethylenediaminetetraacetic acid (EDTA) are used. This would lead us to believe that the reactive species most responsible for ACB degradation in this environment are h^+^ and O_2_^•−^.

Furthermore, it is important to investigate the removal of total organic carbon (TOC) to prevent the production of harmful by-products. Therefore, an analysis was conducted to determine the extent of TOC removal, as depicted in Figure 6c. The study found that the removal effectiveness of TOC rises as the irradiation time increases. After 90 min, the elimination of total organic carbon (TOC) reaches a level of 72.68%, which is slightly lower than the effective rate of deterioration. According to many sources [48,49], the decomposition of aromatic rings in ACB’s structure can potentially convert them into aliphatic acids including acetic acid, carboxylic acid, and oxalic acid. In addition, these feeble acids undergo decomposition into carbon dioxide and water. Hence, the persistence of smaller molecules in the degraded solution can explain the 27% prevalence of TOC after 90 min. Fortunately, these abovementioned acids are less harmful to the ecosystem. The assessment of catalyst stability and reusability is a main parameter in determining its suitability for long-term real-time applications.

The need for the reusability and durability of catalysts for practical applications necessitated the evaluation of five cycles of ACB degradation. Following each reaction, the Ce@MnO_2_ (40:60) catalyst was thoroughly rinsed with distilled water and ethanol using ultrasonic waves. Subsequently, the substance was gathered using centrifugation at a speed of 4000 revolutions per minute for a duration of 5 min. It was then subjected to a drying process at a temperature of 60^o^ C for a period of 12 h in preparation for its further utilization. According to the data presented in Figure 6d, the degradation rates of ACB after each cycle are 92.71%, 89.69%, 87.01%, 84.71%, and 82.72%. These results demonstrate a high level of photocatalytic reusability. A marginal decline may occur due to the loss of photocatalyst during each collection. Furthermore, the XRD patterns of the Ce@MnO_2_ before and after five cycles are displayed in Figure 6e. The diffraction peaks exhibit consistency both prior to and following the process, with no presence of impurities or additional phase peaks. Based on this, it may be inferred that the composite functions as a catalyst with excellent endurance.

**Figure 7:**
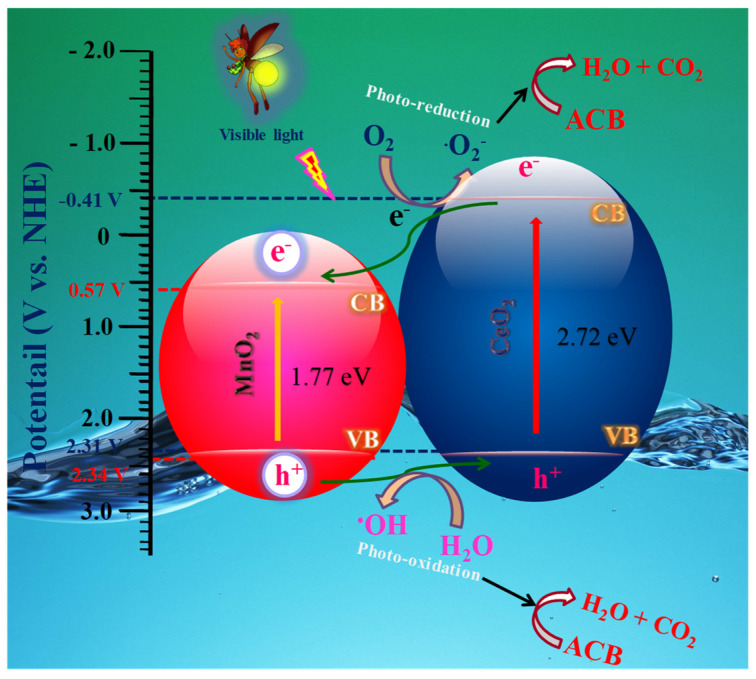
Schematic representation of Acebutolol (ACB) degradation mechanism using Ce@MnO_2_ (40:60) photocatalyst.

The increased photocatalytic process of the Ce@MnO_2_ heterojunction was postulated based on photocatalysts’ band gap structure and the scavengers’ impacts, as shown in Figure 7.

Introducing CeO_2_ and MnO_2_ to visible light can generate electron–hole pairs through irradiation. When an electron was excited into the conduction band, it left behind a hole in the valence band. The electrons (e^−^) and holes (h^+^) in CeO_2_ can be introduced into MnO_2_. Simultaneously, a p-n heterojunction was created between the p-type CeO_2_ and n-type MnO_2_ photocatalysts. During the process, the electrons generated by the photoexcitation are transferred from the p-type CeO_2_ to the n-type MnO_2_, while the holes move from MnO_2_ to CeO_2_. This continues until the Fermi level of the system reaches equilibrium. Subsequently, a localized electron field (E) emerged at the boundary between CeO_2_ and MnO_2_, leading to the efficient segregation of photo-generated electron and hole pairs. The photoexcited electrons subsequently generated O_2_^•−^ and ^•^OH species. Ultimately, all of the active species underwent a reaction with the ACB solution. The p-n heterojunction Ce@MnO_2_ photocatalysts have demonstrated superior photocatalytic efficiency compared to pure CeO_2_ and MnO_2_.

## 3. Materials and Methods

### 3.1. Materials and Reagents

All of the reagents are readily available, have a high analytical purity level, and are ready to use, thus no extra purification steps are needed. The experimental solutions were prepared using double distilled (DD) water.

### 3.2. Preparation Ce@MnO_2_ Nanocomposite

The Ce@MnO_2_ catalyst was prepared using the hydrothermal approach. Approximately 0.5 g of KMnO_4_ was placed into a 100 mL beaker and mixed with 50 mL of deionized water while stirring continuously for 30 min. Subsequently, 6 mL of HCl was added dropwise to the suspension solution mentioned above. Next, a solution containing 0.1 M of Cerium acetate was added to the stirring solution. Following the agitation, the suspension solution underwent a hydrothermal reaction in a 100 mL Teflon lined autoclave at a temperature of 150 °C for a duration of 6 h. Upon the conclusion of the reaction, the samples involved in the reaction were gathered and subjected to a water wash. Subsequently, they were dried at a temperature of 60 °C for a duration of one day. In addition, the catalyst was employed during the annealing process at a temperature of 550 °C for a duration of 3 h. Ultimately, the Pure Ce@MnO_2_ was gathered and employed in further characterization methodologies.

### 3.3. Materials Characterization

The analysis of nanoparticle crystallization was conducted using a (Bruker D8 Advance, Germany) X-ray diffractometer. The JCPDS standards program was utilized to make predictions on the data. The nanocomposite underwent functional group analysis using a PerkinElmer FT-IR spectrometer (Spectrum 100,Waltham, MA, USA). The sample morphology was analyzed using high-resolution transmission electron microscopy (FE-TEM, JEM-2100F, JEOL Ltd., Tokyo, Japan) and scanning electron microscopy (EVO18-CARL ZEISS, Jena, German) with energy-dispersive X-ray Spectra. The Shimadzu UV-2600, (Shimadzu Corporation, Kyoto, Japan) UV-DRS instrument was used to determine the absorbance range and bandgap energy of the photocatalyst. BaSO_4_ was used as a reference material. The absorption spectra of photodegradation were studied using the Hitachi (U-2001, Tokyo, Japan) UV-visible spectrophotometer.

### 3.4. Photodegradation Process

The effectiveness of the photocatalyst was assessed by measuring its ability to degrade Acebutolol (ACB) when exposed to visible light. For every trial, 0.02 g of the photocatalyst were evenly distributed into a 50 mL solution of 10 μM of ACB. Before exposing the mixture to visible light, it was agitated in darkness for 0.5 h to reach a state of adsorption-desorption equilibrium. Throughout the process, 3 mL of solution was extracted at specified time intervals, and the resulting supernatant was recovered using centrifugation. The concentration of ACB was assessed using a UV–vis spectrophotometer. The degradation efficiency was determined using the following formula:

D% = (C_0_ − C_t_)/C_0_ × 100% (3)

where C_0_ represents the initial concentration of the ACB solution and C_t_ represents the concentration of the ACB solution after being exposed to light for varying durations.

## 4. Conclusions

In this study, we successfully synthesized Ce@MnO_2_ catalysts via a simple hydrothermal method and evaluated their photocatalytic performance in degrading the pharmaceutical contaminant Acebutolol (ACB). The Ce@MnO_2_ (40:60) nanocomposite demonstrated a superior degradation efficiency, achieving a remarkable 92.71% reduction in ACB under visible light irradiation within 90 min. This exceptional performance can be attributed to the optimized structural and electronic properties of the Ce@MnO_2_ composite, which facilitated effective photogenerated charge separation and active species generation. The study’s findings underscore the potential of Ce@MnO_2_ nanocomposites as highly efficient, cost-effective, and environmentally friendly photocatalysts for water purification. The excellent reusability and stability of the Ce@MnO_2_ catalyst further enhance its practical applicability, making it a strong candidate for large-scale wastewater treatment processes. Our research introduces a novel and effective approach to addressing pharmaceutical pollution in aquatic environments, highlighting the significant promise of Ce@MnO_2_ photocatalysts in enhancing the quality of wastewater discharged by various industrial sectors. Future studies should focus on scaling up this technology and exploring its application to a broader range of contaminants, paving the way for sustainable water resource management.

## Data Availability

Data are contained within the article.
